# Self-sealing MEMS spray-nozzles to prevent bacterial contamination of portable inhalers for aqueous drug delivery

**DOI:** 10.1007/s10544-022-00628-w

**Published:** 2022-08-05

**Authors:** Torben S. Last, Thomas E. Winkler, Göran Stemme, Niclas Roxhed

**Affiliations:** 1 KTH Royal Institute of Technology, Stockholm, Sweden; 2 Karolinska University Hospital, Bioclinicum, Solna, Sweden; 3 Present Address: Technische Universität Braunschweig, Braunschweig, Germany

**Keywords:** MEMS, Portable inhaler, Drug delivery, Aerosol, Bacterial safety

## Abstract

Pulmonary drug delivery by portable inhalers is the gold standard in lung disease therapy. An increasing focus on environmentally friendly inhalation currently spurs the development of propellant-free devices. However, the absence of propellants in the drug creates a need for suitable sealing systems that can ensure the pathogenic safety of devices. Traditionally, liquid drug inhalers incorporate a spray nozzle and a separate check valve. Here we show a fully integrated MEMS-based spray system for aqueous drug solutions and demonstrate its bacterial safety. The device comprises a thin silicon membrane with spray orifices, which self-seal against a compliant parylene valve seat underneath. This sealing system prevents bacterial ingrowth in its default closed state, while actuation lifts the membrane from the valve seat upon pressurization and sprays an inhalable aerosol from the nozzles. To seal against bacterial contamination effectively, we found that a contact force between the valve seat and the membrane (featuring the spray nozzles) is needed. In our testing, both self-sealing and an otherwise identical unvalved version of the spray chip can be bacterially safe in continued use when thoroughly cleaned of excess fluids and subjected to low bacterial loads for brief periods. However, when directly exposed to $10^{7}$ CFU/ml of our test organism Citrobacter rodentium for 24 h, unvalved systems become contaminated in nearly 90% of cases. In contrast, self-sealing spray chips reduced contamination probability by 70%. This development may enable preservative-free drug formulations in portable inhalers that use propellant-free aqueous drug solutions.

**Supplementary Information:**

The online version contains supplementary material available at 10.1007/s10544-022-00628-w.

## Introduction

**Fig. 1:**
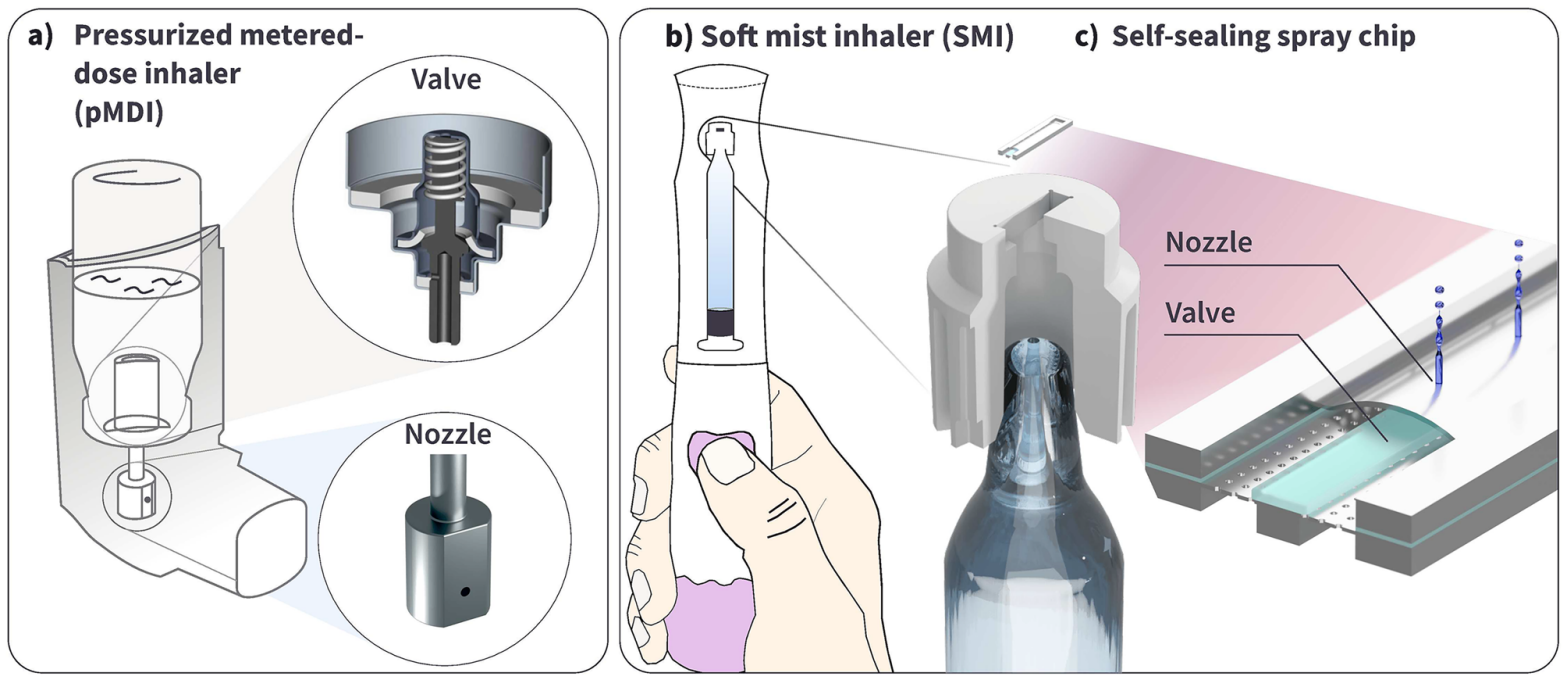
**a** Typical configuration of a Pressurized Metered-Dose Inhaler (pMDI) with the propellant container, metering valve, and spray nozzle unit. The valve graphic is adapted from Stein et al. (2013), **b** Illustration of a Soft Mist Inhaler (SMI), which sprays aqueous drug solutions through a MEMS spray nozzle, **c** 3D illustration of the 3.8, x 1 mm Self-sealing spray chip used in this study. This device combines a check valve and an aerosolization unit into a single chip. The spray nozzle mounts on the tip of a glass syringe, which is pressurized using mechanical springs and actuated at the press of a button to aerosolize 100 µl of a liquid drug

Lung disease is preferably treated using portable inhaler systems (Dolovich and MacIntyre 2000). Multi-dose portable inhalers provide excellent convenience since they can be carried around on-person until needed and allow treatment over an extended period (Navaie et al. 2020). However, their long-term environmental exposure poses an inherent risk for contamination with microorganisms from either the environment or the oral microbiome of the user (Prabhu et al. 2016; Levesque and Johnson 1984; Borovina et al. 2012). Bacterial contamination of inhalation devices is thus a significant concern. Environmental bacterial strains pose a particular threat, as such pathogens have developed mechanisms to bypass the lungs’ protective epithelial resistance mechanisms (Sharma et al. 2020). Pathogens that are transferred to the surface of an inhalation device can ultimately contaminate its drug reservoir (Pierce and Sanford 1973). A spray from a contaminated inhalation device can deliver pathogens to the susceptible lungs of patients on subsequent use; If bacteria get aerosolized in sufficiently small liquid droplets, they can reach terminal lung units and overcome the immune system more easily (Stein and Thiel 2016; Pierce and Sanford 1973).

Two types of portable inhalers currently dominate the market: Dry Powder Inhalers (DPIs) that aerosolize a dry powder formulation, and Pressurized Metered-Dose Inhalers (pMDIs), shown in Fig. 1a), that use gas propellants to spray a liquid and deploy a separate valve and nozzle unit (Agnihotri et al. 2021). However, the greenhouse-gas active propellants of pMDIs have urged the development of more climate-friendly solutions for liquid-formulation inhalers, leading to a third commercially available inhaler type – Soft Mist Inhalers (SMIs) (Wilkinson et al. 2019; Baiker 2015). SMIs have further benefits such as a comparably slow-moving aerosol, which aids drug uptake by the lung, no freeze-effect upon inhalation, which can be uncomfortable to the patient, and broad compatibility towards different drug formulations (Dalby et al. 2004). On the other hand, pMDI devices have a significant benefit in the constantly pressurized drug container, which protects it from pathogenic ingress (Newman 2005).

To realize basic pathogenic safety, SMIs currently on the market have to rely on low concentrations of anti-pathogenic preservatives added to their drug formulations (Koehler et al. 2004). These actively inhibit and/or kill the growth of microorganisms that may have been introduced during multiple actuations of the device (Moser and Meyer 2011). Nasal sprays and medical eyedrops, which also employ aqueous drug solutions, have shown successful transitions toward preservative-free drugs by integrating suitable valve systems into their devices (Birkhoff 2020). However, nasal spray and eyedrops operate at much lower differential pressures, which is less technically challenging to implement compared to SMIs. Here we demonstrate a fully integrated self-sealing MEMS-based spray system and show its performance in resisting bacterial contamination. Critically, we improve upon our first-generation design (Last et al. 2020) with a valve that is not only self-sealing but further retains a non-zero contact force on the valve in the default closed state. We study bacterial ingress into the drug reservoir compared to unvalved controls for two contamination scenarios. A repeated bacterial challenge at low bacterial loads, which we call dynamic testing, and a one-time bacterial challenge at high bacterial load, which we call static testing. To the best of our knowledge this is the first study of this kind performed on liquid-formulation portable inhalers.

## Methodology and study design

### Choice of a challenging organism

**Fig. 2:**
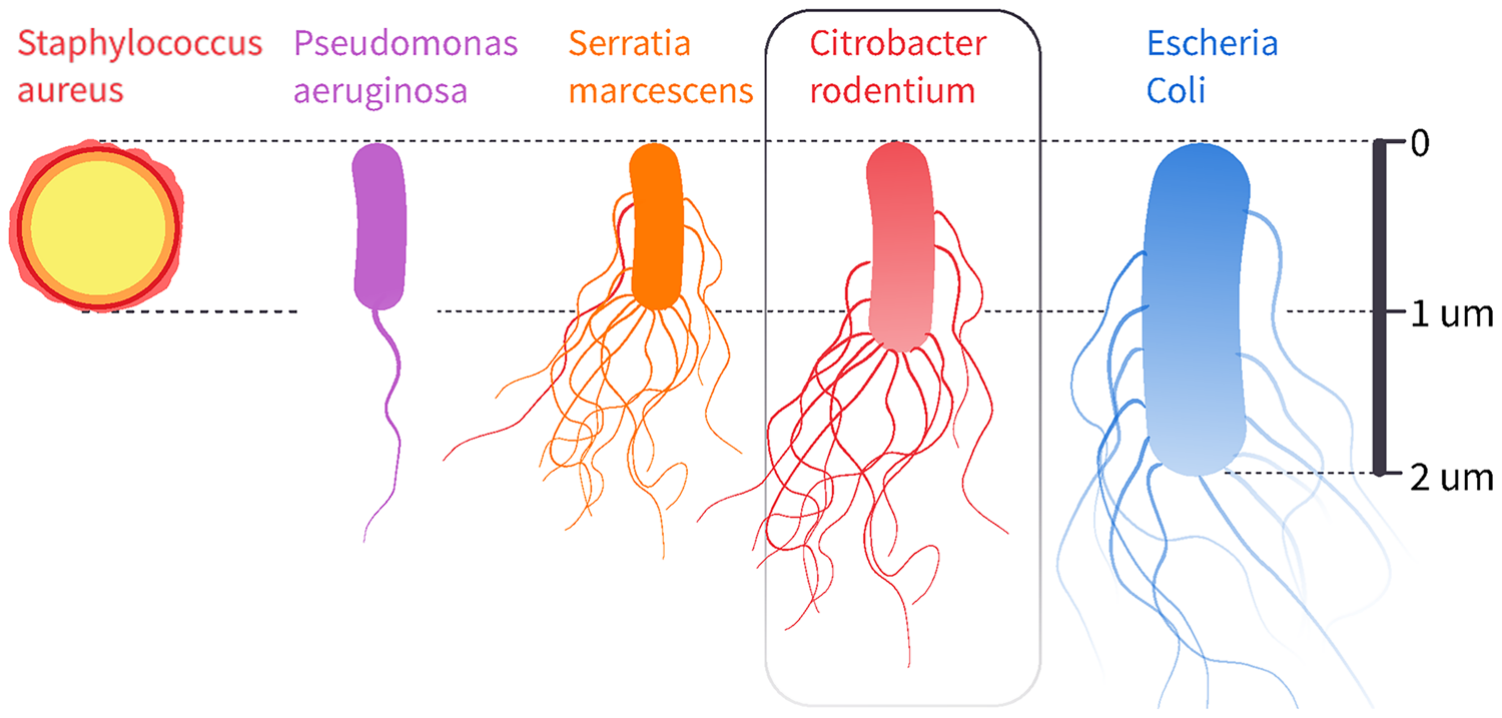
Size comparison and categorization of different bacterial species relevant to inhalation devices, also see S1

To determine a suitable organism to challenge spray chips with, we performed a literature study on bacteria that have previously been collected from inhalers. Relevant bacterial species for inhaler contamination are summarized in S1 and are dominated by gram-negative bacteria, some resistant to antibiotics (Levesque and Johnson 1984; Barnes et al. 1987; Borovina et al. 2012; Jarvis et al. 2014). The collected studies are limited regarding their sample collection. For example population densities of collected specimens were not analyzed. Notably, for the purposes of our present study, the parameters that influence ingrowth may not be the same as those that correlate with surface contamination. Yet, extrapolating from this surface population, we expect that small, motile species have the highest chance of breaching a microbial seal and entering the reservoir. Männik et al. (2009) found that small, motile Escherichia coli bacteria can penetrate microchannels only half their diameter. It is further reasonable to assume that bacteria initially found on the inhaler surface are also capable of at least quiescent or dormant survival, and thus contamination, once inside the reservoir. To challenge our 2 µm diameter nozzle holes (and even smaller gap seal), we selected the gram-negative Citrobacter rodentium. At only around half the size of E. Coli along all axes, C. rod are among the smaller bacterial strains found on inhaler surfaces. Unlike many of the identified strains, C. rod are moreover widely characterized and can be cultured and studied with ease at only biosafety level 1 (ATCC 2021). C. rod are among the organisms collected from inhalers and are especially suitable as an ingrowth test organism due to their high motility, small size, and ease of use. Like E. Coli, they are enteric bacteria and share a core genomic structure common to both commensal and pathogenic strains (Mundy et al. 2005). They have peritrichous flagella and thus have a wide range of motile abilities (Frederiksen 2015). As illustrated in Fig. 2, such flagella are a defining trait of the gram-negative bacteria found on inhalers.

### Chip geometry

**Fig. 3:**
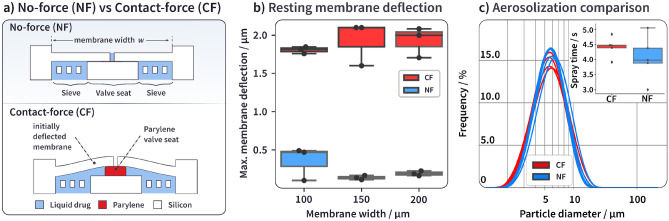
**a** Illustration of the two different chip configurations and their geometric differences, **b** Maximum membrane deflection measurements on the spray chip configurations for different membrane sizes without applied pressure, **c** Aerosolization comparison between NF and CF chip configurations showing a volumetric particle size distribution. The inset shows the spray time for the different configurations for the same measurement

We previously presented a valved spray chip that seals by geometric constraint without contact force (here called a No-force configuration or NF) and that shows a pressure-dependent valving action (Last et al. (2020)). In the present work, we increase sealing performance by introducing a residual contact force (CF) in the default closed state between the valve seat and the membrane featuring the spray nozzles. This contact force is created by an elevated polymer valve seat of parylene (Fig. 3a) alongside the NF design. We chose a valve seat height of 1.9 µm for the CF design. This distance corresponds to 5 bar of pressure being applied to the flat membrane of an NF chip with 150 µm width (Last et al. (2020)). Both chip designs have 60 nozzle holes of 2 µm diameter equidistantly placed on the membrane. To generate pharmaceutically relevant droplet sizes, the spray orifice diameter needs to be roughly 1.5 - 2 µm. The range in which the diameter of the nozzle hole can be changed is therefore tightly restricted, and we have not investigated ingrowth properties of different nozzle diameters. The two chip designs have different membrane deflections at rest (Fig. 3b, raw data in S2), in line with the additional height provided by the parylene layer. The CF chips avoid any gap by sealing against the polymeric valve seat intimately with contact force, while NF chips have a residual membrane deflection of 0.2 - 0.5 µm.

However, channel heights of up to 0.3 µm have previously been shown to prevent the ingrowth of motile Escherichia coli (E. Coli) bacteria in silicon-based devices (Männik et al. 2009). Based on this assumption, the NF chip configuration (at least for 150 µm and wider membranes) should be sufficient to prevent bacterial contamination.

### Test procedure

**Fig. 4:**
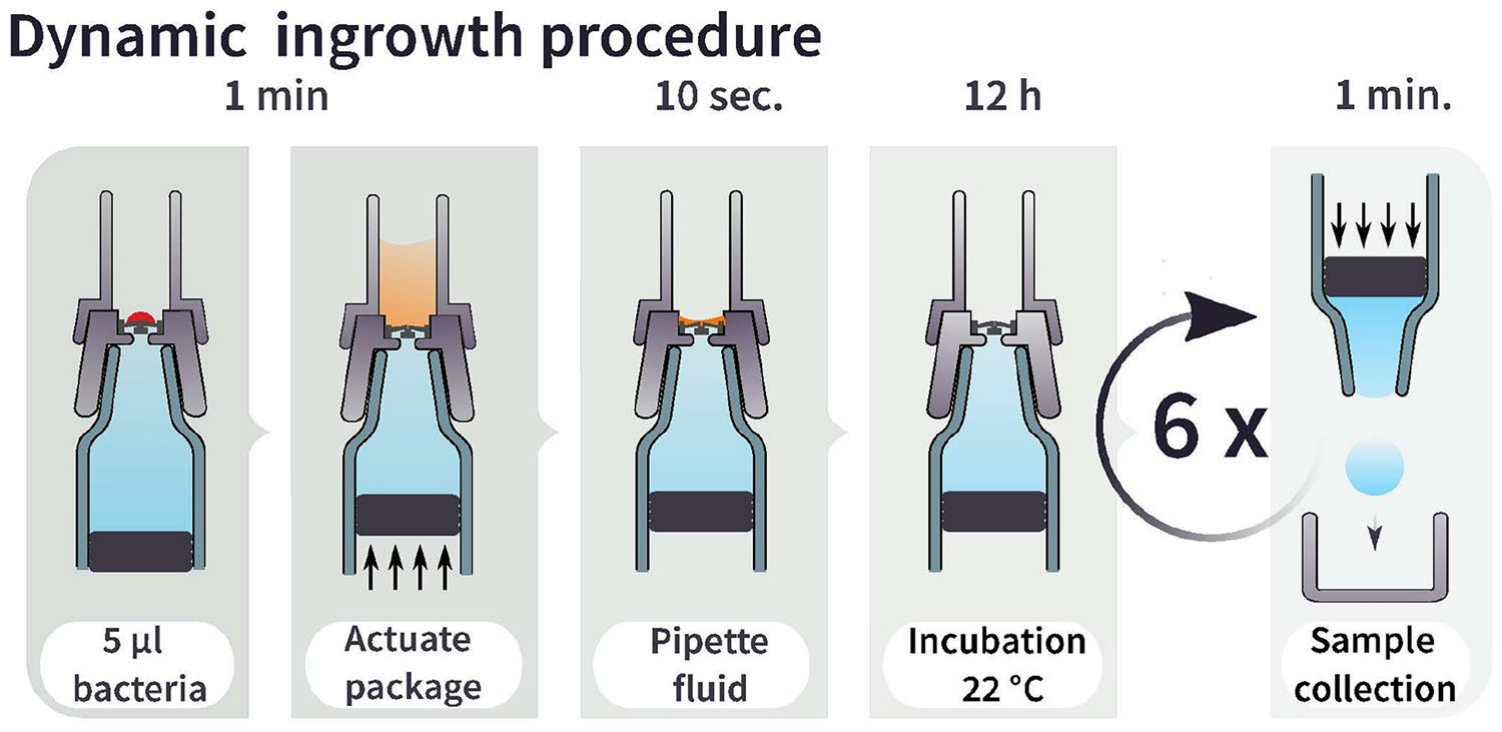
Dynamic ingrowth test protocol, illustrating the application of a 5 µl droplet of C. rod bacterial solution to the spray membrane, followed by the actuation of the plunger. Fluid is then pipetted out of the microwell, and the package is incubated at 22 °C for 12 h. This sequence is repeated six times over the course of three days, after which the fluid from inside the syringe is collected and analyzed

We exposed spray chips to two types of testing conditions: *dynamic* and *static*. In both trials, we use the same syringe packages used as actual drug reservoirs in the Pharmaero SMI portable inhaler platform (Hickey 2019). Phosphate-buffered saline (PBS) is used inside of the syringes as a recovery medium and as a low-nutrient buffer for the bacteria to study penetration rather than proliferation. At the end of the test period, the buffer solution is tested for contamination. The dynamic ingrowth test is set up to simulate a common use pattern for inhalers, where the inhaler is used twice a day at room temperature while repeatably being exposed to typical environmental bacterial loads (Xie et al. 2009). Chips are actuated six times, as illustrated in Fig. 4, and challenged by 5µl droplets (roughly 1 mm diameter) of concentrated bacterial solution at each actuation. This test is similar in scope and methodology to tests performed on medical eyedroppers to evaluate their pathogenic resistance (Birkhoff 2020). However, temperature, time of exposure, bacterial load, and flow conditions can have a strong influence on the number of bacteria that can successfully attach to a surface (Isberg and Barnes 2002), and such tests may underestimate real-world conditions. Therefore, we tried to evaluate a worst-case scenario in our static test, where the spray chip is exposed to extreme conditions for 24 h. Spray chips are actuated once and then subjected to a high load of bacteria, which are allowed to settle on the spray membrane.

**Fig. 5:**
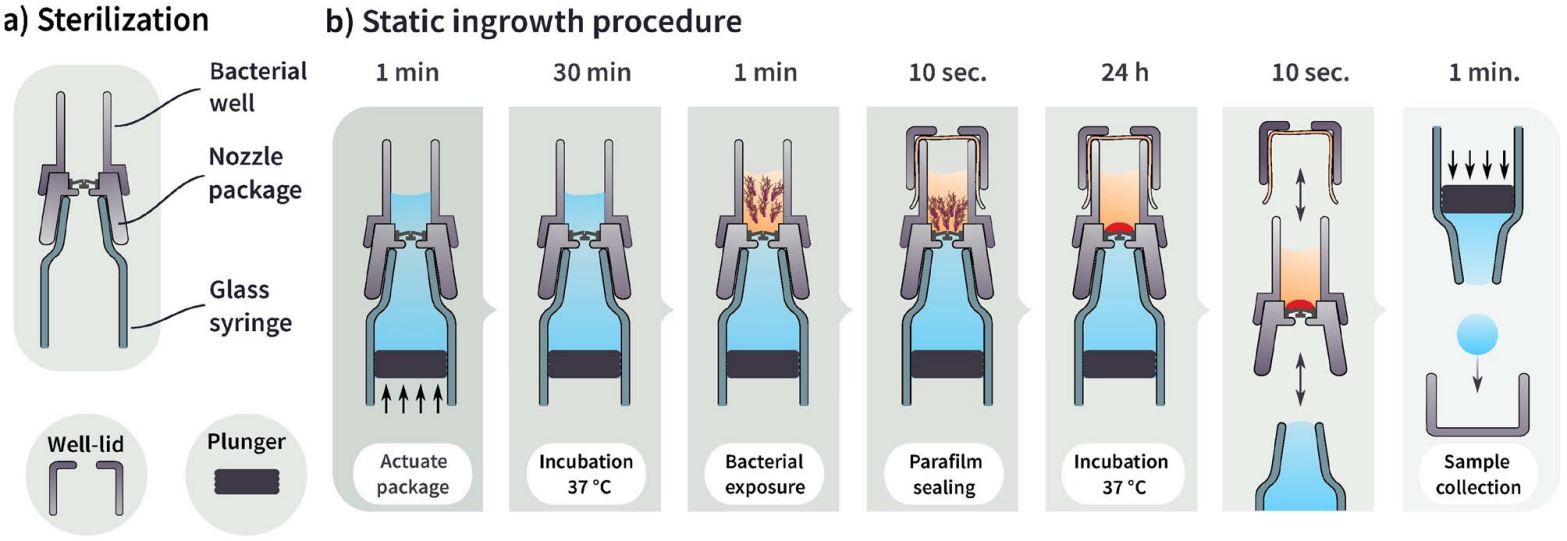
**a** Sterilization of package parts in different units. Bacterial well, nozzle holder, and glass syringe are autoclaved as one glued together package, **b** static ingrowth test protocol starting with applying pressure on the plunger to dispense 100 µl of liquid through the spray chip into the bacterial well. Packages are then stored at 37 °C for 30 m to reach equilibrium conditions. Bacteria are added to the microwell, which is then covered by Parafilm. Packages are incubated at 37 °C for 24 h, before samples from the syringe and the microwell are collected

#### Dynamic ingrowth procedure

In this scenario, illustrated in Fig. 4, the spray chip is repeatedly exposed to a small volume of bacterial solution. The volume and bacterial density commonly created by exhaled droplets due to talking or coughing roughly resembles a 5 µl droplet with a bacterial concentration of $10^7$ CFU/ml (Xie et al. 2009), corresponding to 50.000 bacteria. We exposed spray chips to such droplets of C. rod in PBS, which were placed directly on the membrane with the spray orifices. The syringe’s plunger is actuated using a mechanical fixture, which dispenses 100 µl from the reservoir of the syringe through the spray chip, where it is collected in a 3d-printed microwell on top of the nozzle holder. This fluid is carefully pipetted away, simulating liquid removal as it would happen during a regular spray event. Spray chips are then placed upright in a box, and the packages are incubated at 22 °C for 12 h with gravity causing bacteria to settle on the surface of the spray chip. This procedure is repeated over the course of 3 days, with six actuations of each spray chip. Each of the six times, the spray chips are again challenged by a fresh 5 µl droplet. An equivalent non-sealing spray chip was included as a comparison to the tested CF chips. If the 5µl of bacterial solution contains viable bacteria, these should proliferate and grow in the LB solution.

**Fig. 6:**
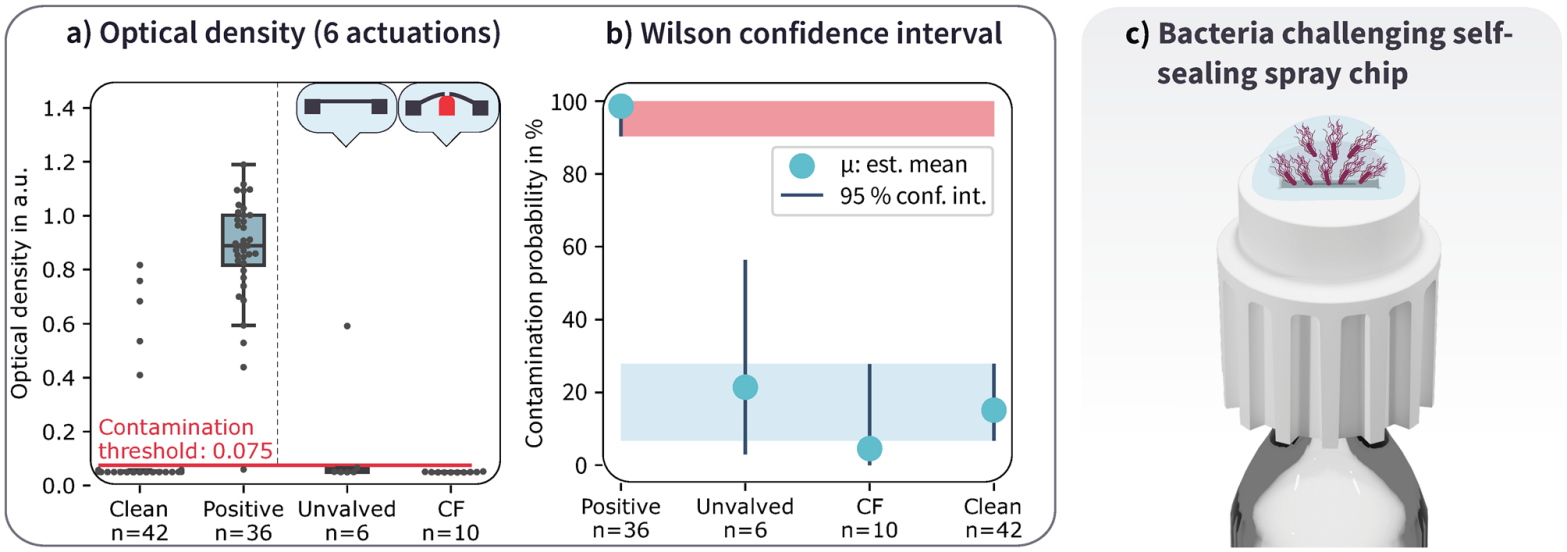
Dynamic ingrowth testing **a** Optical density of cultivated samples after collection, compared to positive and negative controls **b** Confidence intervals for the contamination probability of the different chips under test, **c** Rendered illustration of the placement of a bacteria-containing droplet on the packaged spray chips

#### Static Ingrowth procedure

In the static test, a 30 times higher bacterial load is used to challenge the spray chip compared to the dynamic test. The test was performed for 24 h at an elevated temperature of 37 °C, and bacteria are allowed to settle on the membrane of the spray chip as illustrated in Fig. 5. These testing conditions give the bacteria the chance to form a rudimentary biofilm.

The spray chips with nozzle holes in their membranes (CF, NF, Unvalved) have 100 µl of PBS solution dispensed through the spray chip. The negative control packages (continuous membranes without nozzles) are pressurized to detect leakage. All packages are placed in an incubator at 37 °C for 30 min under sterile conditions to obtain thermal equilibrium. 75 µl of C. rod with $2\,\cdot \,10^{7}$ CFU/ml is pipetted into the microwells. A layer of Parafilm then covers the microwells to prevent drying while enabling oxygen exchange for the bacteria in the well. The packages are then incubated with a bacterial solution in the microwell for 24 h.

## Results & Discussion

### Aerosolization performance

As shown in the inset Fig. 3c), we found the spray performance between CF and NF chips to be indistinguishable within the standard deviation of measurement, both in generated particle sizes as well as in the spray time. This indicates that the opening of the valve is sufficient during operation and does not impede spray performance.

### Dynamic ingrowth testing

A total of 31 spray chips were subjected to dynamic ingrowth testing, out of which 70% of the spray chips made it through the whole cycle of six actuations (the remainder suffered failures unrelated to the design of our spray chips). A procedural graphic, as well as the raw test data, are provided in S3.

**Fig. 7:**
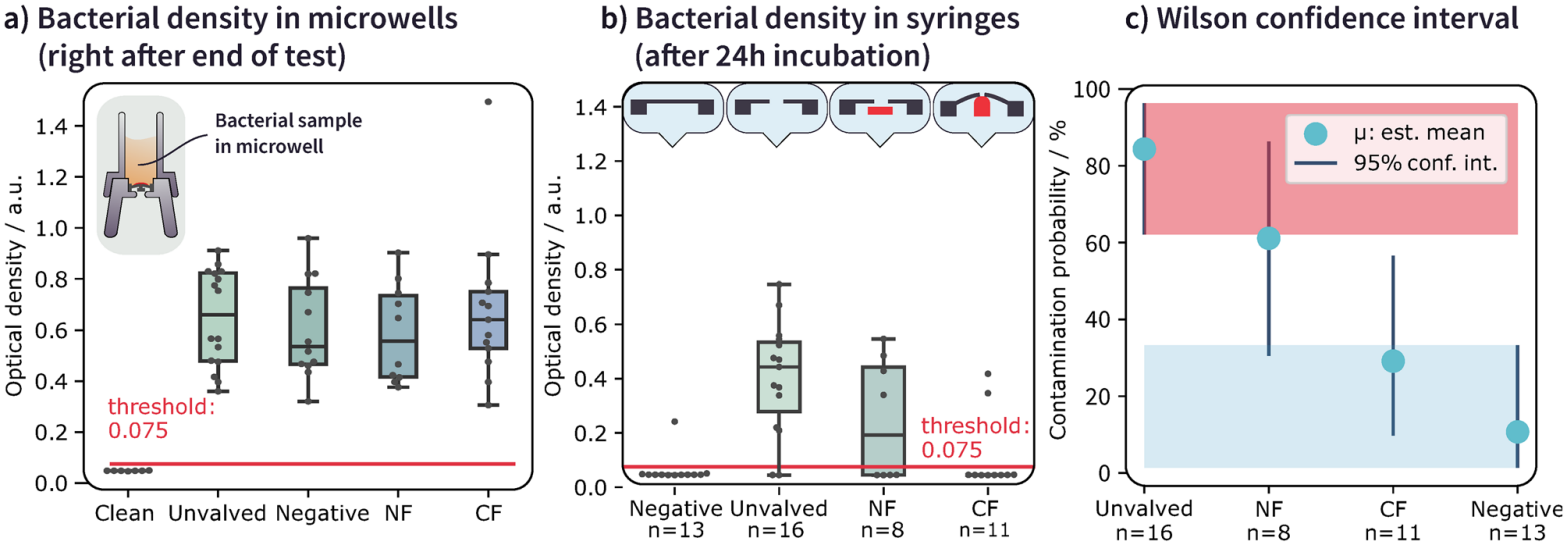
**a** Optical density in the bacterial microwells for the static ingrowth test, collected for the different types of membrane configuration and compared to a clean sample. All samples collected from microwells show high optical density, indicating high bacterial concentrations, after the test. **b** Bacterial density of samples collected from syringes, 24 h after incubation in LB growth medium. Sketches of the membrane configuration are given in the top row as a visual aid for distinguishing the chip designs. **c** 95% confidence intervals for the contamination probability of the different chips under test. The contamination interval of unvalved chips is highlighted in red, the negative control is highlighted in light blue for comparison purposes

35/36 of the positive control samples came back positive, implying that our devices were exposed to viable bacteria at every stage of the test (Fig. 6a), except for a single outlier. 1/6 (16.7%) of unvalved chips were contaminated during the test, while 0/10 (0%) of the CF chips were contaminated. 5 out of the 42 clean control samples were contaminated during testing. Collecting these samples during the test allowed us to restore sterile conditions before collecting fluid from the syringes. After replacing syringe tips, the collected clean samples came back clean again. Since unvalved and CF configuration showed little difference, we omitted NF chips in this test. Looking at the confidence intervals in Fig. 7b), we see that the CF chips lie right within the confidence interval for the unvalved spray chips, and both are well isolated from positive controls but well within the clean control interval. That indicates that both spray chip variants do perform nearly equally well, and the single unvalved sample that came back contaminated is not sufficient evidence to conclude that CF chips perform better. However, given the low applied bacterial load, further testing is needed to quantify the bacterial resistance of the different chip variants.

### Static ingrowth testing

In the static test, the spray chips were actuated once and then exposed to the bacterial solution for 24 h under ideal growth conditions. Figure 7a) shows the bacterial density in the microwell after incubation in the growth medium after the 24 h test period.

In our testing, all positive control tests for dynamic ingrowth testing have come back positive, with high measured optical densities ($\ge \,0.7$), indicating bacterial concentrations in the range of $10^9$ CFU/ml. In Fig. 7b) the optical density of samples collected from the syringes (drug reservoir) is shown. The raw data for samples collected from the microwell and the drug reservoir of the static test is given in S4.

For the negative control, which consists of an unpierced membrane in this test, no bacterial contamination should be possible. However, 1/13 (7%) of chips were contaminated during testing. This may be either due to errors in sample handling or a faulty glue seam around the package. The majority (14/16 or 87%) of the unvalved spray chips were contaminated during the static ingrowth test. Contrary to our expectations, NF chips were not sufficient in hindering ingrowth under our applied test conditions and showed contamination in 4/8 cases (50%). This may be due to the short length of the constriction of the valve seat (15 µm, as the valve seat width is a total of 30 µm) compared to prior publications on spatial constrictions (Männik et al. 2009), where a channel length of 50 µm was used. Further, a membrane structure is not as rigid as an etched channel in a silicon device and may deform when subjected to force. An initial deflection of several hundred nm takes only a few hundred Pascal of pressure acting on the membrane (Last et al. 2020). Bacteria may indeed be able to assert such pressures during ingrowth conditions.

The CF chips showed a significantly lower contamination rate, with only 2/11 chips contaminated. This is even more evident in Fig. 7c) when evaluating the 95% confidence intervals for the measurement data. NF chips show a significant overlap with the unvalved spray chips. On the other hand, we were able to completely isolate the CF chips from the unvalved spray chip contamination rate, which is not the case for NF chips. The valving system, as well as the added residual contact force in the CF chips, are both critical factors in preventing contamination.

## Conclusion

We presented and investigated a fully integrated MEMS-based aerosolization unit featuring self-sealing between the nozzle membrane and valve seat. The spray orifices in the membrane are sealed on nozzle level in the default closed state, leading to minimal dead volume. Two valve-sealing designs were investigated; one design that seals by a default geometric constraint and one novel design which seals by a contact force. Upon actuation at a pressure of 35 bar, the self-sealing chip transfers into an open state and sprays a liquid Rayleigh-jet. In our testing, we could see no difference in spray performance between the two different chip configurations. We tested the bacterial safety of different spray chip configurations by evaluating the contamination probability after bacterial (Citrobacter rodentium) exposure. In a dynamic test, we repeatedly subjected spray chips to a 5 µl droplet over the course of three days. Only one single unvalved chip was contaminated in this test. Due to the low applied bacterial load and the small number of packages tested, we were not able to distinguish between the contamination probabilities of the different chip designs. However, CF chips perform significantly better than a state-of-the-art SMI spray chip when subjected to a 24 h challenge by bacteria at 37 °C. In this test, 14/16 (87.5%) unvalved chips and 4/8 (50%) NF chips failed the test, while only 2/11 (18%) of CF chips became contaminated. When compared to the NF design, our results show that a significant improvement in sealing performance was obtained by introducing residual contact force. Future work on the evaluation of the sealing properties with a broader range of organisms (including gram-positive bacteria and fungi) could further elucidate on the contamination mechanisms. Our small footprint, single-chip sealing system can be manufactured using standard cleanroom procedures cheaply in bulk. This development may enable preservative-free drug formulations in portable inhalers that use propellant-free aqueous drug solutions.

## Materials & Methods

### Fabrication of Self-Sealing Spray Nozzle Chips

The spray chips were fabricated using standard cleanroom manufacturing processes from Silicon on Insulator (SOI) wafers which were bonded using Parylene-C as an adhesive, as described previously (Last et al. 2020). Our SOI wafers had a device layer of 2.5 µm. 60 spray nozzles with a diameter of 2 µm were etched into the device layer of the wafers, which forms the silicon membrane. For contact force chips, a 1.9 µm thick Parylene layer was located on a second SOI wafer which, upon bonding the two wafers, deflects the spray membrane in the normal, unpressurized condition by the height of the Parylene layer. As a result, there is a normal pressure always acting on the spray membrane. Membrane deflection measurements of CF and NF chips with different membrane widths *w* were acquired using white light interferometry on a Wyko NT9300 (Veeco, NY). Apart from its excellent barrier properties and chemical inertness, Parylene-C holds the highest biocompatibility rating for plastics (ISO 10993, USP class VI) (Kim and Meng 2016). It is ideal as a valve-seat material, which needs to be tolerated by a wide range of pharmaceutical drug ingredients without adverse effects. Chips were diced from 100 mm wafers into 3.8 mm by 1 mm chips using a Disco dicing saw.

### Spray testing

We compared the spray performance of NF and CF chips in a Spraytec particle diffraction measurement tool (Malvern Panalytical, Malvern, UK). For more information on the setup, see (Last et al. 2020). One chip of each design was actuated five times using a Pharmaero SMI platform, obtained from SHL Group AB (SHL). Our spray chips were incorporated into this SMI platform, where chips were pressurized with 35 bar, aerosolizing 100 µl of 0.9% saline solution as a model drug. The generated volumetric particle size distributions are measured at an inlet flow rate of 20 slm into the Malvern Spraytec.

### 3D-printing of holding structures for spray chips

Holding structures for the spray chips, illustrated in Fig. 6c), were 3D-printed from Formlabs’ High Temp V2 Resin on a Formlabs 3 printer (3D-Verkstan, Stockholm, Sweden). The high-temperature resin is necessary for subsequent autoclaving. Printed holders were first washed in isopropanol for 20 min and then cured in a Formlabs curing station at 68 °C for 2 h. Spray chips were then glued into these holders using UV-curable Epotek OG198-55 (GA Lindberg ChemTech AB, Stockholm, Sweden). In our testing, the epoxy glue forms a leak-tight seal around the chip. Our holding structure was used both for ingrowth testing as well as the aerosolization comparison in Sect. 6. For the static ingrowth test, packages were sterilized using a Systec Dx 23 autoclave at 121 °C for 20 min.

### Bacterial stock cultivation

We use Citrobacter rodentium (C. rod) (ATCC 51459), a biosafety class 1 organism. C. rod are cultivated in Fisher BioReagents^TM^ Microbiology Media lysogeny broth (LB), Miller (Thermo Fisher Scientific, Stockholm) at 37 °C for 24 h. The bacterial stock solution is diluted down 1/10 with Gibco 14040117 Phosphate-buffered saline with Ca & Mg (PBS) (Thermo Fisher Scientific, Stockholm). This sample is centrifuged twice (10.000 rpm for 5 min) using a VWR Microstar 12 (VWR, Stockholm, Sweden) centrifuge. Each time the supernatant is pipetted away carefully and replaced with PBS. After centrifugation, the optical density of the sample is measured using a Thermo Scientific Multiskan FC (Thermo Fisher Scientific, Stockholm, Sweden), and the corresponding CFU/ml is calculated as described in S5. The sample is then further diluted down with PBS to a bacterial concentration of $10^7$ CFU/ml.

### Bacterial handling

We cultivated 1 ml of collected liquid from the SMIs drug reservoir in growth media for 24 h. If a viable bacterium is present in the solution it would start to colonize, resulting in an exponential growth that can be readily quantified after 24 h using an optical density measurement. Samples with an optical density smaller than 0.075 after 24 h incubation were counted as clean. Samples with higher optical densities were counted as contaminated.

#### Dynamic ingrowth control samples

As a clean control, we pipetted 200 µl of PBS solution into an incubation tube with 3 ml LB growth medium to certify sterile handling of packages during the ongoing test. We pipetted 5 µl of bacterial solution into 3 ml of LB growth medium as a positive control.

#### Static ingrowth control samples

As a negative control, we include silicon chips without nozzle holes in the test, which are otherwise packaged identically to the valved spray nozzle chips. As a positive control, we collect approximately 175 µl of bacterial fluid from the microwells, cultivate the sample and then measure optical density, as described in Sect. 5.5.3.

#### Sample collection

After incubation, the PBS solution was collected from the syringes. In the case of the static ingrowth test, bacteria from microwells were collected as well, using a pipette. Before actuating their plungers, the syringes were wiped with an ethanol cloth on their Luer connection. The plunger was then pressed, emptying the contents of the syringe into a well-plate. In the case of the static ingrowth test, 2 ml of solution were collected. Due to the number of actuations during the dynamic ingrowth test cycle, only 1.4 ml were collected from the syringe after dynamic ingrowth testing. For both tests, we pipetted 1 ml from each collected sample contained in the well plate and then placed these samples into an incubation tube that contained 3 ml of LB growth medium. These tubes were then incubated for 24 h. Optical density was measured using a 96-well-plate in the Thermo scientific Multiscan FC on five samples of 200 µl from each cultivation tube, and optical densities were averaged from those five samples.

### Statistical analysis

As motivated in the introduction, a single CFU in the drug reservoir can be enough to void the pathogenic safety of an inhalation device. Therefore, we performed tests that come back either contaminated (1) or clean (0). Statistically, this represents a Bernoulli process with only two outcomes (1) or (0). This discreteness of results makes the analysis of any Bernoulli process deceptively tricky (Song et al. 2009). We assume the true contamination rate of chips *p* depends only on the geometry of the tested chips by keeping all the background variables constant. The observed contamination rate

1 $$\begin{aligned} p_o = \frac{n_{pos}}{n_{pos}+n_{neg}} \end{aligned}$$

calculates the ratio of how many of the tested chips come back positive. $p_o$, however, may differ from *p*. The question then becomes how often one has to repeat the test in order to be confident that the observed contamination rate $p_o$ is close enough to the true contamination rate *p*. This question can be answered using a suitable confidence interval, which gives an upper and lower limit in which p lies, given $p_o$ and the number *n* of tests performed. Out of various confidence estimations for the Bernoulli process, we have chosen a 95% Wilson interval (Wilson 1927), which provides particularly robust confidence intervals for a small number of trials (Song et al. 2009). Wilson intervals were calculated using the statsmodels module in Python (Seabold and Perktold 2010).

## Supplementary Information

Below is the link to the electronic supplementary material.

Supplementary file1 (PDF 1.11 PDF)

## Data Availability

Raw data is available in the supplementary information.
