# Supplementary material for: Self-sealing MEMS spray-nozzles to prevent bacterial contamination of portable inhalers for aqueous drug delivery
Source: Biomed Microdevices. 2022 Aug 5;24(3):25. doi: 10.1007/s10544-022-00628-w (PMC9355933; doi:10.1007/s10544-022-00628-w)
Supplement: Supplementary file 1 — Supplementary file1 (PDF 1.11 PDF) [file 10544_2022_628_MOESM1_ESM.pdf]

Biomedical Microdevices manuscript No.
(will be inserted by the editor)

Self-sealing MEMS nozzles to prevent bacterial contamination of portable inhalers for aqueous drug delivery

Supplementary Information

Torben S. Last, Thomas E. Winkler, Göran Stemme · Niclas Roxhed

Received: date / Accepted: date

S 1 Aggregated data on bacteria found on inhalation devices

Most devices analyzed in the following studies are stationary inhalers, such as nebulizers. Some of the studies were performed in a clinical setting, where inhalation devices are subject to sterilization procedures. Surface contamination of portable inhalation devices is rather common as shown by Levesque and Johnson (1984), who analyzed the portable inhalers of 12 children and found all mouthpieces of the analyzed inhalers positive for bacterial growth. 67 % of these inhalers were never washed. The cultured organisms of this study were not speciated. In a study performed by Barnes et al (1987), the stationary home nebulizers of 50 patients were investigated, most of which showed severe pathogenic contamination. Interestingly, this is the only study in which gram-positive organisms dominated the collected bacteria. A study performed by Borovina et al (2012) was conducted in 2008 in Australia on a total of 45 pMDIs. 53 % of these devices showed visible debris on the inside of the mouthpiece, and 80 % of the devices were colonized with microorganisms. Some of the collected bacteria were shown to be antibiotically resistant. The most recent study by Jarvis et al (2014) concluded that even recommended nebulizer washing techniques might not eradicate microorganisms on inhalation devices, but only found 30 % of the analyzed inhalers to be colonized.

Bacteria collected from inhalation devices are aggregated in Table 1. Even though the presented studies have some limitations regarding sample collection, gram-negative environmental species are the dominant source of bacterial contamination on inhalation devices.

Torben S. Last, Göran Stemme and Niclas Roxhed
KTH Royal Institute of Technology
E-mail: roxhed@kth.se

Thomas E. Winkler
Technische Universität Braunschweig
E-mail: thomas.winkler@tu-braunschweig.de

S 2 Raw maximum membrane deflection data

We have measured membrane deflection for three different membrane sizes, which have all come from the same wafer that our tested chips originate from, using a *Wyko NT 9300* optical profilometer. CF chips have a membrane with an intentionally deflected membrane (by the Parylene gasket) of 1.9 µm. This leads to an initial force that is acting between valve seat and membrane at all times, even when no pressure is applied to the chip. The NF chips should show no membrane deflection other than residual deflection due to compressive stress. For the NF chip design we measure deflections at rest in the order of 100 – 500 nm. For the CF chips we measure values close to 1.9 µm, the height of the Parylene gasket between valve seat and membrane. For the 100 µm membranes we measure slightly smaller maximum deflection values, possibly due to Parylene compression.

Table 2 contains the deflection data for the NF and CF chips, measured on samples that originate from the same wafer, using white light interferometry.

S 3 Dynamic ingrowth supplement

A total of 31 spray chips were subjected to dynamic ingrowth testing, out of which four were removed from the test due to a broken membrane and five more due to a leaking Luer connection. In total, 70 % of the spray chips made it through the whole cycle of six actuations. A detailed illustration of how many chips made it through

Table 1 Bacterial types collected from inhalers. Fungi were found and collected in Borovina et al (2012) and may play a further role in pathogenic contamination of inhalers. Inhalers that were analyzed in a clinical setting were subject to sterilization procedures, while home-use inhalers may or may not have been sterilized by the user. Collected bacteria are split into gram-positive and gram-negative groups and publications are listed by the year of publication.

Publication	Setting	Device Type	Collected gram-negative bacteria	Collected gram-positive bacteria
Levesque and Johnson (1984)	Home-use	pMDI	cultured organisms were not speciated	cultured organisms were not speciated
Barnes et al (1987)	Clinical	Nebulizer	Pseudomonas, Acinetobacter, Serratia marcesans (S. marcesans), Flavobacteum	Staphylococcus albus, Diphtheroids, Micrococcus sp, β Haemolytic streptococcus, Streptococcus viridans, Staphylococcus aureus (S. aureus)
Borovina et al (2012)	Home-use	pMDI	Staphyllococcus spp, E. Coli, Resistant E. Coli, Resistant Klebsiella, Enterobacter and Serratia, Haemophilus spp, Pseudomonas aeruginosa,	S. aureus, Enterococcus spp,
Jarvis et al (2014)	Clinical	Nebulizer	Pseudomonas aeruginosa, Escherichia coli (E. Coli), Serratia marcesans (S. marcesans), Klebsiella pneumonia, Enterobacteriaceae	S. aureus

Table 2 Raw membrane deflection data comparing NF and CF chips. Columns contain the membrane width of the spray chip, membrane length is 2 mm. Deflection values for the different membrane widths are given in µm.

Contact-force	100um	150um	200um
no	0.46850	0.11025	0.2250
no	0.48940	0.13375	0.1626
no	0.10030	0.16550	0.1860
yes	1.75950	1.59980	1.7055
yes	1.81470	2.10040	2.0830
yes	1.84775	2.10025	1.9985

the different steps is given in Fig. 1, which shows the testing procedure for dynamic ingrowth testing.

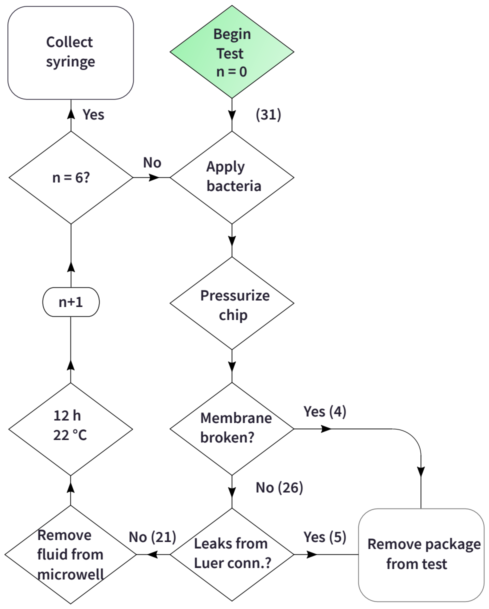

Fig. 1 Dynamic ingrowth protocol, with the number of chips at each step given in brackets.

S 3.1 Raw dynamic ingrowth data

The raw optical density data for the dynamic ingrowth test is given in Table 3.

S 4 Static ingrowth supplement

We removed packages that showed leakage (either around the glue connection of the chip or the Luer connection

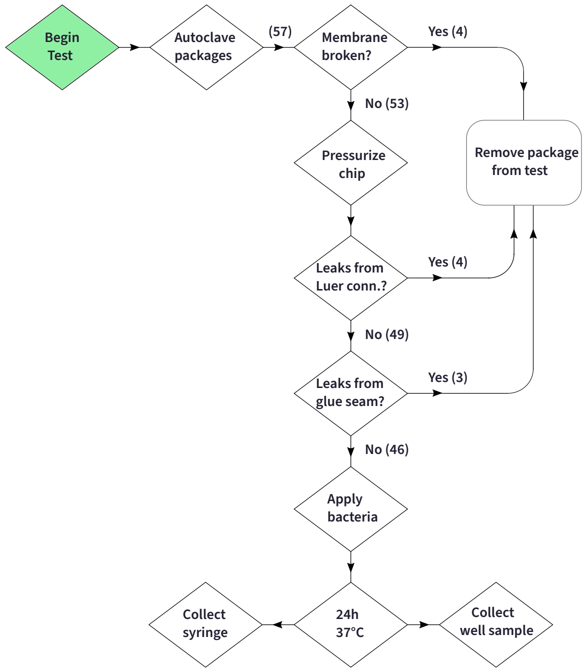

Fig. 2 Static ingrowth protocol, with the number of chips at each step given in brackets.

to the syringe) and were therefore prone to bacteria contaminating the syringe without passing the sealing system. We further removed packages with damaged membranes after the autoclavation step. In total, 11 out of 57 chips were sorted out before going through the 24h ingrowth procedure. Further, a single chip was sorted out post-experiment as no bacteria could be cultivated from its bacterial well. Fig. 2 shows the testing procedure for static ingrowth testing.

While performing the ingrowth test we encountered several reasons for spray chips to fail the ingrowth test that do not bear on the validity of the spray chips themselves.

- Membrane damaged during autoclavation of spray packages.
- Test package leaks from the Luer connection.
- Test package leaks from the glue seam around the spray chip.

Figure 2 shows the ingrowth procedure, with failures documented for each step. Table 4 shows the raw data of collected optical densities from syringe packages. Testing was performed over two months with 5 separate testing runs. In total 48 packages were tested, all chips had a membrane width of 200 µm

We further collected the bacterial density in the bacterial microwells which are loacted on top of the spray chip. This measurement serves two functions: first, we assure that bacteria do survive for the time of the ingrowth test in the microwell. Second, this indicated just how many bacteria

A single sample out of 48 samples was excluded from the dataset, as no bacteria could be cultivated from the bacterial well. This data is collected in Table ??.

S 5 CFU calculation from OD

The number of colony-forming unit (CFU) that are present in a growth medium based on measured optical density (OD) were determined using plating at different Citrobacter rodentium (ICC 168) OD. OD was acquired from 250 ml samples, collected into 96-well plates. Equation 1 is valid in an optical density range between $0.07 < OD < 1.5$.

$$\frac{CFU}{\mathrm{ml}} = \frac{OD(620\,\mathrm{nm}) - 0.0601}{1.44439 \cdot 10^{-10}} \tag{1}$$

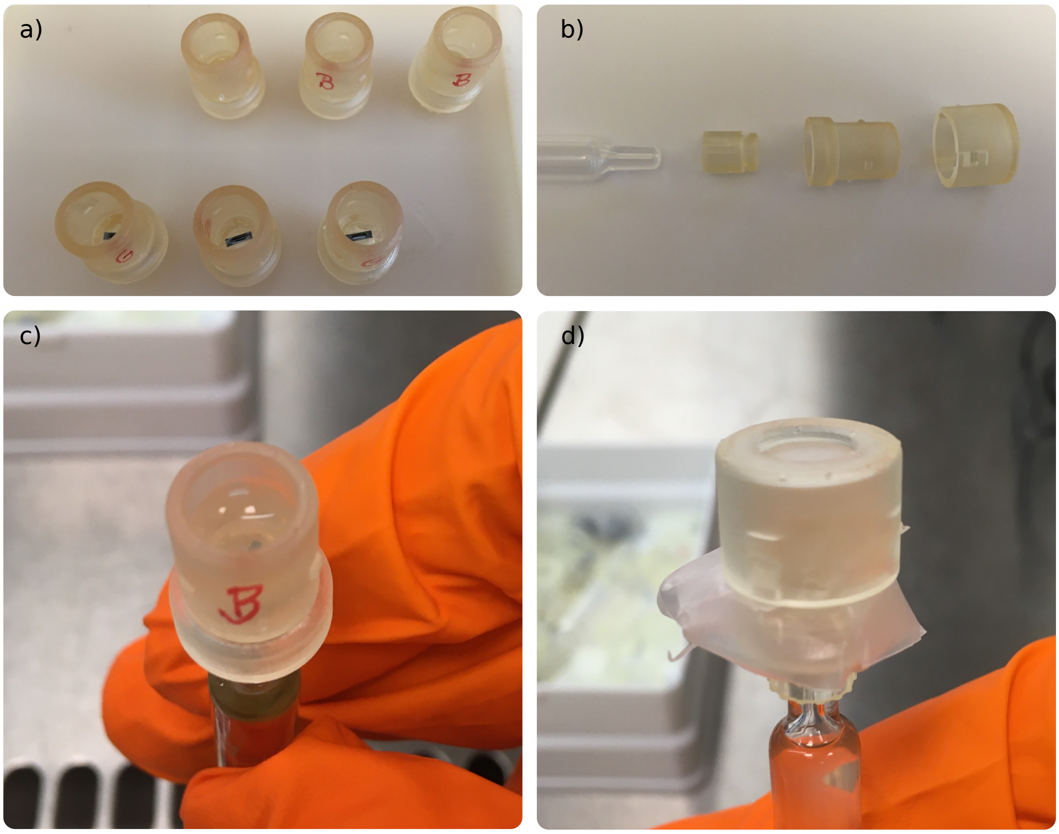

Fig. 3 a) Glued packages, where the spray chip is glued into the nozzle holder and the microwell is glued on top. Looking from the top, the black rectangle in the middle is the spray chip, b) from left to right shows the syringe with the Luer connection, then the nozzle package, the microwell and the lid, c) shows the microwell filled with a bacterial solution after removing the Parafilm cover from the syringe, which is visualized in d).

Table 3 Raw optical density data for the dynamic ingrowth test.

Date	Neg	Pos	Unvalved	CF 150 1.8
2021-04-29	0.0500	0.8704	0.5918	0.0494
2021-04-29	0.0500	1.0396	0.0496	0.0500
2021-04-29	0.0518	0.9780	0.0500	0.0498
2021-04-29	0.0576	0.9640	0.0498	0.0508
2021-04-29	0.0490	0.8884	-	-
2021-04-29	0.0492	0.8892	-	-
2021-04-29	0.0508	0.7962	-	-
2021-04-29	0.0500	0.7396	-	-
2021-04-29	0.0500	1.1892	-	-
2021-04-29	0.0494	1.0014	-	-
2021-04-29	0.0512	0.5292	-	-
2021-04-29	0.0496	0.5942	-	-
2021-04-29	0.0504	-	-	-
2021-04-29	0.0516	-	-	-
2021-06-07	0.0520	0.6872	-	0.0486
2021-06-07	0.0528	0.7696	-	0.0486
2021-06-07	0.0532	0.0596	-	0.0520
2021-06-07	0.0510	0.4390	-	0.0488
2021-06-07	0.0520	0.8234	-	-
2021-06-07	0.0512	0.8592	-	-
2021-06-07	0.0520	0.9110	-	-
2021-06-07	0.0526	1.0270	-	-
2021-06-07	0.0542	0.8466	-	-
2021-06-07	0.0538	0.8330	-	-
2021-06-07	0.0524	1.0968	-	-
2021-06-07	0.0508	1.0946	-	-
2021-06-07	0.0502	-	-	-
2021-06-07	0.0504	-	-	-
2021-06-20	0.0498	1.0122	0.0512	0.0488
2021-06-20	0.0518	1.0928	0.0660	0.0486
2021-06-20	0.0532	0.8566	-	-
2021-06-20	0.0566	0.8966	-	-
2021-06-20	0.0490	0.9846	-	-
2021-06-20	0.0494	1.0016	-	-
2021-06-20	0.4098	0.8724	-	-
2021-06-20	0.6838	0.8538	-	-
2021-06-20	0.7578	1.1162	-	-

Table 4 Raw optical density data for the static ingrowth test. Clean samples are 1ml PBS in 3ml LB. Negative tests have a chipt with a membrane that lacks nozzle holes, Unvalved spray chips do not feature a valve system but the same nozzle array configuration as NF and CF chips: 60 nozzles with 1.8 µm, NF chips feature no force on the valve seat while CF chips have a valve seat thats extended by 1.9 µm.

Date	Clean	Negative	Unvalved	NF	CF
2020-08-21	0.0456	0.0460	0.3680	0.3400	-
2020-08-21	0.0460	0.0456	0.3750	0.4284	0.0454
2020-08-21	-	0.0460	0.3382	0.0454	-
2020-08-28	0.0450	0.0480	0.7472	0.0444	0.0448
2020-08-28	0.0450	0.0444	0.6706	0.0448	0.0442
2020-08-28	-	-	0.5236	0.5464	0.0444
2020-08-28	-	-	0.0450	-	0.4178
2020-08-28	-	-	0.5446	-	0.0450
2020-08-28	-	-	0.4762	-	-
2020-08-28	-	-	0.5584	-	-
2020-09-08	0.0470	0.0446	0.2198	-	-
2020-09-08	0.0480	0.0462	0.0446	-	-
2020-09-08	-	0.0506	0.2090	-	-
2020-09-08	-	0.2418	-	-	-
2020-10-04	0.0490	0.0460	0.4700	0.4846	0.0448
2020-10-04	-	0.0460	0.4430	0.0452	0.0452
2020-10-04	-	0.0462	-	-	0.3462
2020-10-04	-	-	-	-	0.0460

Table 5 Raw optical density data from the static ingrowth test showing bacterial density in the microwells. Microwell solution was incubated for 24 h in LB, and the resulting optical density of the solution is shown here.

Date	Clean	Unvalved	Negative	NF	CF
2020-08-21	0.0478	0.4166	0.4352	0.4234	-
2020-08-21	0.0480	0.3964	0.3206	0.3760	0.3058
2020-08-21	-	0.3600	0.4760	0.3968	-
2020-08-28	0.0480	0.7548	0.7472	0.8010	0.6412
2020-08-28	0.0480	0.7994	0.6706	0.9026	0.5522
2020-08-28	-	0.5660	-	0.7456	0.8956
2020-08-28	-	0.5666	-	-	1.4950
2020-08-28	-	0.8292	-	-	0.7852
2020-08-28	-	0.8570	-	-	-
2020-08-28	-	0.7758	-	-	-
2020-09-08	0.0492	0.4802	0.5164	-	-
2020-09-08	0.0478	0.4760	0.5548	-	-
2020-09-08	-	0.5334	0.4610	-	-
2020-09-08	-	-	0.4672	-	-
2020-10-04	0.0460	0.9108	0.8208	0.7034	0.5806
2020-10-04	-	0.8286	0.8188	0.6476	0.7076
2020-10-04	-	0.8208	-	-	0.6950
2020-10-04	-	-	-	-	0.7512

Table 6 Excluded data from the collected samples of the static ingrowth test

Date	Sampletype	CF
2020-08-21	Bacterial well	0.0476
2020-08-21	Syringe sample	0.0456

References

Barnes KL, Clifford R, Holgate ST, Murphy D, Comber P, Bell E (1987) Bacterial contamination of home nebuliser. Br Med J (Clin Res Ed) 295(6602):812

Borovina LR, Tremellen KE, Walker MP, Hawley TM, Horgan AR, Grant GD, King MA (2012) The microbial contamination of pressurised metered-dose inhalers anonymously sourced from the South-East Queensland Australia community population: Microbial contamination of metered-dose inhalers. International Journal of Pharmacy Practice DOI: 10.1111/j.2042-7174.2011.00168.x

Jarvis S, Ind PW, Thomas C, Goonesekera S, Haffenden R, Abdolrasouli A, Fiorentino F, Shiner RJ (2014) Microbial contamination of domiciliary nebulisers and clinical implications in chronic obstructive pulmonary disease. BMJ Open Respiratory Research 1(1):e000018, DOI: 10.1136/bmjresp-2013-000018

Levesque KA, Johnson CE (1984) Bacterial contamination of pressurized inhalers. Drug Intell Clin Pharm 18(9):735–737

All links were last followed on November 13, 2021.
